# Community pharmacists’ interest in and attitude to pharmacy practice research in Ethiopia: A cross-sectional study

**DOI:** 10.1371/journal.pone.0178919

**Published:** 2017-06-15

**Authors:** Akshaya Srikanth Bhagavathula, Eyob Alemayehu Gebreyohannes, Begashaw Melaku Gebresillassie, Daniel Asfaw Erku, Chernet Tafere Negesse, Yared Belete Belay

**Affiliations:** 1 Department of Clinical Pharmacy, University of Gondar-College of Medicine and Health Sciences, School of Pharmacy, Gondar, Amhara, Ethiopia; 2 Department of Pharmaceutical Chemistry, University of Gondar-College of Medicine and Health Sciences, School of Pharmacy, Gondar, Amhara, Ethiopia; 3 Department of Formulation Research and Development, Addis Pharmaceutical Factory, Adigrat, Tigray, Ethiopia; 4 Department of Pharmacoepidemiology and Social Pharmacy, Mekelle University, Mekelle, Tigray, Ethiopia; CANADA

## Abstract

Pharmacy practice-research became an important component in the pharmacy practice. However, limited studies were conducted in sub-Saharan Africa to understand the pharmacists’ interest and attitude towards pharmacy practice-research. We aimed to assess the community pharmacists’ interest and attitude towards pharmacy practice-research in Ethiopia. A cross-sectional survey was conducted among community pharmacists in eight major cities in Ethiopia. A validated 25-item self-administered questionnaire covering interest and attitude related to pharmacy practice-research was distributed. Responses were analysed using descriptive and inferential statistics. A total of 389 community pharmacists responded to the survey (response rate- 88.4%). Most of community pharmacists showed a high level of interest and positive attitude in being involved in all aspects of pharmacy practice-research. The median summary score for interest and attitude were 38 (IQR 20–40) (range possible 10–50) and 30 (IQR 18–39), respectively. Sixty-seven percent of the respondents thought about being involved in research, felt research is important for their career (57.6%), confident to conduct the research (56.2%), and agreed that research is a part of pharmacy practice (48.5%). However, only forty-six percent agreed that they underwent research training. A multivariate analysis showed that females were more interested in pharmacy practice research than males [AOR: 1.50, 95% CI: 0.99–2.27; *p*<0.05]. Community pharmacists showed high interest towards several areas of research competencies and demonstrated positive attitude towards pharmacy practice-research. Our findings suggest that providing research training to community pharmacists may contribute in undertaking research activities and build the research capacity in Ethiopia.

## Introduction

In pharmacy, rapid advances in technology, education and practice have fostered community pharmacists’ interest in extending their practice beyond their traditional role in the healthcare system. In order to provide these extended services, they need to adopt and establish evidence-based practice. Evidence-based medicine (EBM) is defined as “the conscientious, explicit and judicious use of current best evidence in making decisions about the care of current individual patients” [1]. Every year, several rigorous research conducted to address clinical questions about patient outcomes and cost-effective health care. These substantive finding helped to answer the questions from accessing, collating and interpreting results to provide best treatment for a particular population suffering with particular condition. One classical example would be the early studies demonstrating the value of aspirin in prevention of cardiovascular diseases in diabetic patients [2]. However, implementing evidence-based pharmacy practice is not easy, as pharmacists must have solid research skills.

Several studies have identified key factors such as a lack of knowledge, awareness, experience, skills, and understanding as some of the barriers to practicing EBM [3–8]. In addition, earlier work from developed countries such as the United Kingdom (UK), Canada, Australia, and Qatar has shown that although pharmacists may be aware of research, they are less likely to participate in research activities [3, 5,7–10]. This situation was also noted among physicians and nurses [11, 12]. However, surveys conducted with UK pharmacists report that 32 to 48% were interested in participating in research [13–15]. Similarly, in Australia, pharmacists with some research experience (77%) were more likely to undertake future research than those who had never participated (34%) [16].

Community pharmacists can make a significant contribution to future research initiatives from small scale local projects to large scale treatment effectiveness of new services [17]. Professional pharmacy organizations in developed countries have devoted much effort to developing and supporting pharmacy practice research [18]. Further, developing countries such as Qatar [19, 20], Saudi Arabia [21] and Thailand [22] also assessed the extent of interest and exposure to research processes among pharmacists [19–22]. All these studies aimed to investigate the importance of pharmacy practice research in different practice levels as a potential area of improvement in the current era. Till date, no studies have addressed the need of pharmacy practice research in community pharmacists and pharmacy technicians in Ethiopia. Due to its widespread implications, this study assessed the level of interest and attitude of community pharmacists and pharmacy technicians in a sub-Saharan country: Ethiopia. The aim of this study is, therefore, to assess community pharmacists’ and pharmacy technicians’ interest in, and attitude towards pharmacy practice research in Ethiopia.

## Materials and methods

### Design

In this cross-sectional survey, a questionnaire was distributed to all community pharmacists and pharmacy technicians practicing in community medicine retail outlets (CMROs) in Ethiopia from September–December 2015. The CMROs in Ethiopia are divided into pharmacy and drug stores based on the kind of medications they are supposed to dispense and the qualification of service providers. Pharmacies run only by a pharmacist having a qualification of a university degree or above and drug stores run by pharmacy technicians with qualification of diploma in pharmacy. As the roles and responsibilities of pharmacy technicians in pharmacy practice and pharmacy-based research is significant in Ethiopia, we included pharmacy technicians in the survey. A simple random sampling technique was applied and stratified into two city administrations (Dire Dawa and Addis Ababa), three historically advantaged regions (Gondar, Jimma, and Mekelle) and three historically disadvantaged regions (Adama, Hawassa, and Dessie). Each community pharmacist was directly approached at their place of work and given 20–30 minutes to complete the questionnaire.

### Sample size

The single proportion formula was used to estimate the study sample size [23]. Based on the Federal Ministry of Health (FMOH) Health Sector Development Program (HSDP) IV (2010–2015), there were a total of 661 active community pharmacists at the end of 2010 [24]. Accordingly, 440 participants were selected and the questionnaires were equally distributed across the eight cities and regions as there was no available data on the proportion of pharmacists and pharmacy technicians currently practicing in each of the cities and regions.

### Questionnaire

We used a validated questionnaire developed in the *Stewart et al*. [25] study related to pharmacy practice research among hospital pharmacists in Qatar. It was pretested in all areas of pharmacy practice and appropriate modifications were made. The questionnaire consisted of 25 items that were divided into three sub-sections as follows: Part 1 related to sociodemographic and practice information (5 items). Part 2 consisted of closed, 5-point Likert scale (1 = no interest, 2 = little interest, 3 = some interest, 4 = moderate interest, 5 = very interested) statements related to community pharmacists’ interest in research activities (10 items). Part 3 contained 10 closed items related to research and were assessed using a 5-point Likert scale (1 = strongly agree to 5 = strongly disagree) (S1 Questionnaire). Scores for research interest (range 10–50) and attitude (range 10–50) were summed. We used median scores from the data to establish a cut-off score as there were no previous guidelines on how to interpret these scores. Accordingly, scores greater than ≥38 (research interest) and ≥30 (attitude) were considered as positive, <38 was consisted as negative for interest, and <30 negative for attitude. On the Likert scale, five out of the ten attitudinal statements were positively worded and the remainders were negatively worded (2, 4–6 and 9). These negative statements were reverse scored so that higher scores reflected a more positive attitude.

### Data analysis

Responses from each site were manually entered into SPSS software (Cary, NC version 21.0) and were double-checked for accuracy. Descriptive and inferential analyses were performed for sociodemographic, attitude and practice information. The mean, standard deviation, median, interquartile range (IQR) and percentages were computed for all interest and attitude statements. A bivariate analysis was carried out, and variables with a *p*-value less than 0.2 were included in a multivariate logistic regression analysis. Odds ratios with a 95% confidence interval (95% CI) were also computed, along with the corresponding *p*-value.

### Ethical clearance

The study protocol was reviewed and approved by the Institutional Research Board of the School of Pharmacy at the University of Gondar, Ethiopia. The purpose and importance of the study was explained, and written consent was obtained from each study participant. Confidentiality was maintained by not disclosing personal information, and questionnaires were anonymized.

## Results

Out of 440 community pharmacists who were approached, 389 completed the survey (88.4% response rate). Among the study participants, 244 (62.7%) were males, with a mean age of 29.8 years (SD ± 7.6 years). Two hundred and eighty (72%) of the respondents held Bachelor’s degrees in pharmacy (B. Pharm) and 56.3% had less than five years’ experience in community pharmacy practice. More than two-thirds worked in independent pharmacies (35.7%) or drug stores (33%). More than half of participants were from Amhara (25.4%; Dessie and Gondar) and Oromia regions (25.4%; Jimma and Adama), followed by Mekelle (14.1%), Addis Ababa (11.6%) and other cities (Table 1).

**Table 1:** Demographic characteristics of community pharmacists in Ethiopia (N = 389).

Characteristics	Frequency (%)
*Mean Age (years)*	29.8±7.6 (SD)
*Gender*	
Male	244 (62.7)
Female	145 (37.3)
*Level of pharmacy education*	
Diploma (D. Pharm)	64 (16.5)
Bachelors (B. Pharm)	280 (72)
Postgraduate (MSc.)	45 (11.6)
*Work experience*	
< 5 years	219 (56.3)
> 5 years	170 (43.7)
*Type of Pharmacy*	
Independent pharmacy	139 (35.7)
Drug store	128 (32.9)
Chain pharmacy	122 (31.4)
*Pharmacy location (region)*	
Mekelle (Tigray)	55 (14.1)
Jimma (Oromia)	54 (13.9)
Dessie (Amhara)	51 (13.1)
Gondar (Amhara)	48 (12.3)
Hawassa (South Ethiopia)	46 (11.8)
Addis Ababa (Capital, city administration)	45 (11.6)
Adama (Oromia)	45 (11.6)
Dire Dawa (City administration)	45 (11.6)

SD: Standard deviation

Responses to questions about interest in research are summarized in Table 2. Most respondents expressed their interest in all aspects of pharmacy practice research, specifically: ‘research advances within the field’ (70%; n = 272); ‘generating research ideas’ (64.3%; n = 250); ‘analysis and interpretation of results’ (62%; n = 24); ‘giving an oral presentation’ (e.g., national or international conferences) (60.1%; n = 234); ‘reviewing the scientific literature’ (60.1%; n = 234); ‘using qualitative research methods’ (59.3%; n = 231); ‘writing and publishing research in academic journals’ (58.3%; n = 227); and ‘writing research proposals’ (56.8%; n = 221). The median score for interest was 38 (IQR 20–40) (possible range 10–50).

**Table 2:** Community pharmacists’ interest in research activities (N = 389).

Research interest	No interest = 1, n (%)	Little interest = 2, n (%)	Some interest = 3, n (%)	Moderate interest = 4, n (%)	Very interested = 5, n (%)	Median (IQR)
Research advances in my field	16 (4.1)	41 (10.5)	60 (15.4)	111 (28.5)	161 (41.4)	4 (2–4)
Generating research ideas	11 (2.8)	51 (13.1)	77 (19.8)	129 (33.2)	121 (31.1)	4 (2–4)
Finding relevant literature	11 (2.8)	56 (14.4)	88 (22.6)	123 (31.6)	111 (28.5)	4 (2–4)
Systematically reviewing literature	20 (5.1)	54 (13.9)	102 (26.2)	109 (28.0)	104 (26.7)	4 (2–4)
Writing a research proposal and protocol	33 (8.5)	59 (15.2)	76 (19.5)	112 (28.8)	109 (28.0)	4 (2–4)
Using quantitative research methods (e.g. RCTs, cohort studies, surveys, questionnaires)	25 (6.4)	60 (15.4)	89 (22.9)	119 (30.6)	96 (24.6)	4 (1–4)
Using qualitative research methods (e.g. focus groups, interviews)	19 (4.9)	58 (14.9)	81 (20.8)	110 (28.3)	121 (31.1)	4 (2–4)
Analyzing and interpreting results	27 (6.9)	46 (11.8)	75 (19.3)	121 (31.1)	120 (30.8)	4 (2–4)
Giving an oral presentation (e.g. national or international conference)	54 (13.9)	46 (11.8)	54 (13.9)	111 (28.5)	124 (31.9)	4 (3–4)
Writing and publishing research in academic journals	49 (12.6)	37 (9.5)	76 (19.5)	116 (29.8)	111 (28.5)	4 (2–4)

IQR = Interquartile range

Median and interquartile ranges for attitude scores are shown in Table 3. Overall, more than half of respondents had positive attitude, with a median of 30 (IQR 18–39), in a potential range of 10–50. With respect to questions about research involvement, 66.8% (n = 260) of respondents had thought about being involved in research; more than half felt research was important for their career (57.6%; n = 224); were confident in their ability to conduct research (56.2%; n = 219); and agreed that research was a part of pharmacy practice (48.5%; n = 189). However, only 45.7% (n = 178) agreed that they had received training in research. More than fifty percent disagreed with the statements: ‘research is of little importance in Ethiopia’ (62%; n = 241); ‘research is of little importance for community pharmacists’ (58.6%; n = 228); ‘research is irrelevant to their profession’ (55.7%; n = 217); ‘I have no time to think about research’ (55.2%, n = 215); and ‘research is more suitable for academics than community pharmacists’ (44.7%, n = 174) respectively.

**Table 3:** Community pharmacists’ attitude towards research activities (N = 389).

Attitude	Strongly disagree = 1, n (%)	Disagree = 2, n (%)	Unsure = 3, n (%)	Agree = 4, n (%)	Strongly agree = 5, n (%)	Median (IQR)
Being involved in research is important to my career	52 (13.4)	35 (9.0)	41 (10.5)	110 (28.3)	150 (38.6)	4 (2–4)
Research is of little importance to me*	145 (37.3)	83 (21.3)	38 (9.8)	78 (20.1)	45 (11.6)	2 (1–3)
I feel that it is my professional duty to be involved in research	59 (15.2)	48 (12.3)	58 (14.9)	118 (30.3)	106 (27.2)	4 (3–4)
Research is of little relevance to community pharmacists'*	126 (32.4)	91 (23.4)	43 (11.1)	82 (21.1)	47 (12.1)	2 (2–4)
Research is of little importance in Ethiopia*	162 (41.6)	79 (20.3)	25 (6.4)	77 (19.8)	46 (11.8)	2 (1–4)
Research is more suited to academics than community pharmacists*	81 (20.8)	93 (23.9)	66 (17.0)	89 (22.9)	60 (15.4)	3 (2–4)
I have research training	54 (13.9)	67 (17.2)	90 (23.1)	112 (28.8)	66 (17.0)	3 (2–4)
Involvement in research is a part of my practice	43 (11.1)	73 (18.8)	84 (21.6)	109 (28.0)	80 (20.6)	3 (2–4)
I do not have time to think about research*	100 (25.7)	115 (29.6)	39 (10.0)	81 (20.8)	54 (13.9)	2 (1–4)
I am confident that I can conduct research	47 (12.1)	58 (14.9)	65 (16.7)	123 (31.6)	96 (24.7)	4 (2–4)

IQR = Interquartile range

*negative attitude statements

Table 4 shows the associations between sociodemographic and practice information, and interest and attitude. A multivariate logistic regression found that only sex was significantly associated with interest. Females were more likely to be interested in research activities than males [AOR: 1.50, 95% CI: 0.99–2.27]. Those with more than five years’ experience were twice as likely to be interested in research activities [AOR 2.05, 95% CI: 0.70–1.57]. With regard to attitude, there was no significant association for sex, educational qualifications and years of experience in both bivariate and multivariate logistic regressions.

**Table 4:** Bivariate and multiple logistic regression analysis (N = 389).

Variable	Category	Median		COR (95% CI)	AOR (95% CI)
**Interest (range 10–50)**		**< 38**	**≥ 38**		
Sex	Male	108	136	1	1
	Female	79	66	1.521 (1.002–2.309)	1.507 (0.997–2.278)*
Postgraduate qualification	No	169	175	1	1
	Yes	18	27	0.675 (0.353–1.292)	0.690 (0.367–1.300)
Work experience	< 5 years	104	115	1	
	> 5 years	83	87	1.167 (0.771–1.768)	2.055 (0.706–1.575)
**Attitude (range 10–50)**		**< 30**	**≥ 30**		
Sex	Male	117	127	1	
	Female	73	72	1.090 (0.720–1.651)	0.667 (0.354–1.255)
Postgraduate qualification	No	172	172	1	
	Yes	18	27	0.667	0.350–1.272
Work experience	< 5 years	108	111	1	
	> 5 years	82	88	1.019 (0.674–1.539)	0.958 (0.641–1.430)

COR = crude odds ratio

AOR = adjusted odds ratio, 95%

CI = confidence interval

*significant at *p* < 0.05

## Discussion

To the best of our knowledge, this study is the first to investigate interest in, and attitudes to research among community pharmacists in Ethiopia. Our results show that community pharmacists in Ethiopia have a positive interest and attitude towards pharmacy practice research. Overall, they recognize its value in advancing their career, and express a high level of interest in research activities. In general, respondents who expressed a positive attitude were more likely to be involved in it. Pharmacy practice research is an important way to generate new knowledge and improve pharmacists’ skills using evidence-based practice and rational decision-making in patient care. Community pharmacists play a crucial role in research as they are directly involved in patient care and are better placed to implement evidence-based pharmaceutical care in the community.

Research has shown that in developed countries, a large proportion of pharmacists express an interest in pharmacy practice research, but their involvement in research activities is limited. Similarly, in our study, a high number of community pharmacists agreed that research was important in their practice, but only a limited number (46%) had research training. This reflects their desire to contribute to research that can advance the practice in Ethiopia. However, lack of adequate research training and skills are potential barriers that have already been identified in several previous studies [6,8,16,26,27].

A substantial proportion of the community pharmacists who participated in our study had graduate level training and showed a high level of interest in research activities such as analyzing results, reviewing the literature, qualitative research, writing manuscripts, publishing in journals, and writing protocols. This implies that pharmacists with a graduate level of education consider research as an important part of their career. This should be compared to several previous studies conducted in Australia, the UK, and Qatar where a large number of non-postgraduates showed little, if any interest at all, in conducting research [3,9,25]. The possible explanation for this difference in our study may be due to the integration and promotion of pharmacy practice research in the undergraduate curriculum in 2008. Furthermore, in Ethiopia, undergraduates benefit from the same facilities as more advanced students. These factors are key to creating a research environment, encouraging a research culture and supporting pharmacy practice research.

In our study, majority of community pharmacists expressed a positive attitude towards pharmacy practice research. This reflects their interest in integrating research into their professional life and practice. Community pharmacists are better placed to engage in research that will ultimately have an impact on practice. About 57% of participants reported that engaging in research is a professional duty, and nearly half believed that research is part of pharmacy practice. Similar findings were noted by *Elkaseem et al*. in their study of Qatari pharmacists–almost all participants agreed that it was their duty to contribute to pharmacy practice research [8].

Over half (56%) of our community pharmacists believed that they were competent to conduct research. These numbers are lower than those reported by *Elkaseem et al*. [8] and *Awaisu et al*. [18] in their studies on Qatari hospital pharmacists (70%) [25], but higher than in *Perreault et al*.’s study of critical care pharmacists in Canada (51%) [5], and *Kanjanrach et al*.’s (>50%) study of hospital pharmacists in Thailand [6]. Similar numbers have been reported in previous studies, and limited exposure to pharmacy practice research is perceived to be the barrier [8, 28, 29]. National and international organizations should support Ethiopian pharmacists, who already possess a positive attitude to research, in overcoming these barriers and engaging in research that will improve patient outcomes.

No significant association was found between a positive attitude to research and any of the sociodemographic or practice-related variables. This suggests that all respondents give equal importance to research activities and supporting research. However, in *Stewart et al*.’s [25] study on Qatari hospital pharmacists only those with postgraduate qualifications were significantly interested in participating in research. Therefore, the aspirations of individuals must be taken into consideration to overcome the belief that research is only for postgraduates. Future studies could investigate the barriers and outcomes for different categories of pharmacy professionals. Finally, qualitative research based on in-depth interviews would help to understand the real-life barriers faced by community pharmacists in Ethiopia.

The study has some limitations that should be considered while interpreting the results. Firstly, it was a cross-sectional study conducted in eight Ethiopian cities and the findings cannot be generalized to other cities in the country. Second, our use of a self-administered questionnaire that relied upon respondents providing honest answers and the fact that the questionnaire was not specifically validated in Ethiopian setting might subjected to social desirability bias as some respondents may have provided more extreme responses than others, due to their motivations and beliefs, and might subjected to social desirability bias., and their answers might be subject to recall bias.

## Conclusion

A large proportion of community pharmacists in Ethiopia showed a high level of interest in several areas of research and a positive attitude towards pharmacy practice research. Female pharmacists were significantly more interested in participating in research than their male counterparts. These findings suggest that providing training to community pharmacists may be useful in encouraging them to undertake research activities and develop research capacity to implement evidence-based practice in Ethiopia.

## Supporting information

S1 Questionnaire Study questionnaire. (DOCX)

S1 Dataset Data underlying this study. (SAV)
