# Supplementary material for: Community pharmacists’ interest in and attitude to pharmacy practice research in Ethiopia: A cross-sectional study
Source: PLoS One. 2017 Jun 15;12(6):e0178919. doi: 10.1371/journal.pone.0178919 (PMC5472273; doi:10.1371/journal.pone.0178919)
Supplement: S1 Questionnaire — (DOCX) [file pone.0178919.s001.docx]

**University of Gondar**

**College of Medicine and Health Sciences, School of pharmacy**

**Consent Information sheet**

My name is __________________________. I am here on behalf of Gondar University research group staffs. They are conducting a research on ‘*Community Pharmacists’ Interest in and Attitude to Pharmacy Practice Research in Ethiopia: A Cross-Sectional Study*’ Their research project was approved by the Research Ethics committee of Gondar University to conduct this study. Your participation is purely based on your willingness. You have the right to choose not to take part in this study. If you choose to take part, you have the right to stop at any time.

The estimated time to finish the questionnaire is about 20-30 minutes. The study will explore your attitude and interest towards research activities. The information that you provide will be kept confidential by using only code numbers and locking the data.

Based on the understanding of the information I gave you, are you willing to participate in this study?

1. Yes
2. No

**Part 1: Socio-demographic characteristics**

1. Age ______
2. Sex male female
3. Qualification: non-pharmacist undergraduate pharmacist postgraduate pharmacist
4. Time since working as a community pharmacist : <5 years >=5years
5. Type of pharmacy Independent pharmacy drug store Chain Pharmacy

**Part 2: Questions on interest towards research activities:** 1-No interest, 2-Little interest, 3-Some interest, 4- Moderate interest, 5-Very interested

|  | **Questions** | **1** | **2** | **3** | **4** | **5** |
| --- | --- | --- | --- | --- | --- | --- |
| 1 | Research advances within my field |  |  |  |  |  |
| 2 | Generating research ideas |  |  |  |  |  |
| 3 | Finding relevant literature |  |  |  |  |  |
| 4 | Systematically reviewing literature |  |  |  |  |  |
| 5 | Writing a research proposal and protocol |  |  |  |  |  |
| 6 | Using quantitative research methods  (e.g. RCTs, cohort studies, surveys, questionnaires) |  |  |  |  |  |
| 7 | Using qualitative research methods (e.g. focus groups,  interviews) |  |  |  |  |  |
| 8 | Analysing and interpreting results |  |  |  |  |  |
| 9 | Giving an oral presentation (e.g. national or international conference) |  |  |  |  |  |
| 10 | Writing and publishing research in academic journals |  |  |  |  |  |

**Section 3: Questions related to attitudes towards research aspects**

5-Strongly agree, 4-Agree, 3-Unsure, 2- Disagree, 1-Strongly disagree

|  | **Questions** | **1** | **2** | **3** | **4** | **5** |
| --- | --- | --- | --- | --- | --- | --- |
| **1** | Being involved in research is important to my career |  |  |  |  |  |
| 2 | Research is of little importance to me |  |  |  |  |  |
| **3** | I feel that it is my professional duty to be involved in research |  |  |  |  |  |
| 4 | Research is of little relevance to community pharmacists' |  |  |  |  |  |
| 5 | Research is of little importance in Ethiopia |  |  |  |  |  |
| 6 | Research is more suited to academics rather than community pharmacists' |  |  |  |  |  |
| **7** | I already underwent research training courses |  |  |  |  |  |
| **8** | Involving in research is a part of my practice |  |  |  |  |  |
| 9 | I don't have time to think about research |  |  |  |  |  |
| **10** | I am confident to conduct research |  |  |  |  |  |

**Thank you for your participation!!!**
